# Disparities in School Connectedness, Unstable Housing, Experiences of Violence, Mental Health, and Suicidal Thoughts and Behaviors Among Transgender and Cisgender High School Students — Youth Risk Behavior Survey, United States, 2023

**DOI:** 10.15585/mmwr.su7304a6

**Published:** 2024-10-08

**Authors:** Nicolas A. Suarez, Lindsay Trujillo, Izraelle I. McKinnon, Karin A. Mack, Bridget Lyons, Leah Robin, Michelle Carman-McClanahan, Sanjana Pampati, Krista L.R. Cezair, Kathleen A. Ethier

**Affiliations:** ^1^Division of Adolescent and School Health, National Center for Chronic Disease Prevention and Health Promotion, CDC; ^2^Epidemic Intelligence Service, CDC; ^3^Division of Injury Prevention, National Center for Injury Prevention and Control, CDC; ^4^Division of Violence Prevention, National Center for Injury Prevention and Control, CDC

## Abstract

Transgender high school students (those whose gender identity differs from their sex assigned at birth) experience disparate health outcomes and challenges in school, including violence and discrimination, compared with cisgender students (those whose gender identity aligns with their sex assigned at birth). Until recently, population-based data describing the experiences of transgender students and students questioning whether they are transgender (questioning) have been limited. In 2023, the national Youth Risk Behavior Survey assessed transgender identity, providing the first nationally representative data about transgender students. This report describes the demographic characteristics of transgender and questioning high school students and examines differences in the prevalence of experiences of violence, poor mental health, suicidal thoughts and behaviors, school connectedness, and unstable housing among transgender, questioning, and cisgender high school students nationwide. In 2023, 3.3% of U.S. high school students identified as transgender, and 2.2% identified as questioning. Transgender and questioning students experienced a higher prevalence of violence, poor mental health, suicidal thoughts and behaviors, and unstable housing, and a lower prevalence of school connectedness than their cisgender peers. Compared with 8.5% of cisgender male students, 25.3% of transgender students and 26.4% of questioning students skipped school because they felt unsafe. An estimated 40% of transgender and questioning students were bullied at school, and 69% of questioning students and 72% of transgender students experienced persistent feelings of sadness or hopelessness, a marker for experiencing depressive symptoms. Approximately 26% of transgender and questioning students attempted suicide in the past year compared with 5% of cisgender male and 11% of cisgender female students. Intervention opportunities for schools to create safer and more supportive environments for transgender and questioning students can help address these disparities. The findings of this report suggest that more effort is necessary to ensure that the health and well-being of youths who are socially marginalized is prioritized.

## Introduction

Gender refers to the socially constructed norms and expectations imposed on persons according to their designation as male or female sex at birth. Gender identity refers to a person’s sense of self and personal experience of gender. Transgender persons are those persons whose gender identity differs from their sex assigned at birth, whereas cisgender describes persons who identify with the gender aligned with their sex assigned at birth (https://www.who.int/health-topics/gender). Transgender students experience multiple health disparities compared with cisgender students (*1*). Gender identity development is a fundamental part of adolescence; transgender and questioning youth who do not conform to social expectations of gender might experience gender dysphoria, discrimination, or violence. Transgender and questioning students face unique challenges at school, including being unable to use bathrooms or play on sports teams matching their gender identity, being misgendered (i.e., addressed by the wrong name by teachers and peers), and otherwise being unable to express themselves in a way consistent with their gender identity (*2*). Negative experiences at school, including harassment and bullying, contribute to environments where transgender students do not feel safe and supported (*2*). Feelings of school connectedness (i.e., the belief held by students that adults and peers in the school care about them, their well-being, and their success) also might be diminished among transgender students. School connectedness has been linked to positive health outcomes into adulthood and is a protective factor for adolescents facing stress, adversity, or marginalization (*3*,*4*). Housing is a key social determinant of health that influences adolescent health outcomes, and CDC recognizes the importance of safe, healthy housing as part of the agency’s broader health equity strategy (*5*).

Population-based data on the experiences of transgender and questioning students have been limited. In 2023, the national Youth Risk Behavior Survey (YRBS) assessed transgender identity in the United States for the first time. This report provides the first nationally representative estimates of transgender identity among U.S. high school students and examines disparities among experiences of school connectedness, housing instability, violence, mental health, and suicidal thoughts and behaviors comparing transgender, questioning, and cisgender students. Professionals in public health, education, and government, as well as persons and families seeking to support youths in their lives can use these data to understand the experiences and challenges related to health and well-being faced by transgender and questioning students nationwide and address the need to develop strategies that prevent disparate experiences and outcomes for these populations.

## Methods

### Data Source

This report includes data from the 2023 YRBS (N = 20,103), a cross-sectional, school-based survey conducted biennially since 1991. Each survey year, CDC collects data from a nationally representative sample of public and private school students in grades 9–12 in the 50 U.S. states and the District of Columbia. Additional information about YRBS sampling, data collection, response rates, and processing is available in the overview report of this supplement (*6*). The prevalence estimates for transgender identity for the overall study population and by sex, race and ethnicity, grade, and sexual identity are available at https://nccd.cdc.gov/youthonline/App/Default.aspx. The full YRBS questionnaire, data sets, and documentation are available at https://www.cdc.gov/yrbs/index.html. Institutional review boards at CDC and ICF, the survey contractor, approved the protocol for YRBS. Data collection was conducted consistent with applicable Federal law and CDC policy.*

### Measures

YRBS measures and analytic coding are available (Table 1). A single item assessing transgender identity was developed by CDC survey methodologists and external researchers. In 2018, the item was cognitively tested with high school students and found to be understood as written. The question reads, “Some people describe themselves as transgender when their sex at birth does not match the way they think or feel about their gender. Are you transgender?” Students who responded, “Yes, I am transgender,” were categorized as transgender, and students who responded, “I am not sure if I am transgender,” were categorized as transgender or questioning. Students who responded, “No, I am not transgender,” were assumed to be cisgender. Students who responded, “I do not know what this question is asking,” and students who skipped the question were excluded from analyses. Demographic measures included sex (female or male), race and ethnicity (American Indian or Alaska Native [AI/AN], Asian, Black or African American [Black], Native Hawaiian or other Pacific Islander [NH/OPI], White, Hispanic or Latino [Hispanic], or multiracial [selected >1 racial category]) (persons of Hispanic or Latino origin might be of any race but are categorized as Hispanic; all racial groups are categorized as non-Hispanic), grade (9, 10, 11, or 12), and sexual identity (heterosexual, gay or lesbian, bisexual, questioning [I am not sure about my sexual identity/questioning], or described in some other way [I describe my identity some other way]). Cisgender students were further disaggregated by sex. This question does not specify sex assigned at birth, and it is possible that transgender or questioning students might have responded to this question differently than cisgender students. For this reason, transgender and questioning students are not further disaggregated by sex for analysis of health behaviors and experiences. Sex is reported for transgender and questioning students for descriptive purposes (Table 2). Because small numbers of transgender and questioning students identified as AI/AN, Asian, or NH/OPI, data from these three racial groups were suppressed. The health behaviors and experiences examined in this report represent key indicators for adolescents, including an important protective factor for adolescent health and well-being (school connectedness), and a social determinant of health (housing).

**TABLE 1:** Questions, response options, and analytic coding for select health risk behaviors among high school students — Youth Risk Behavior Survey, United States, 2023

Variable	Question	Response option	Analytic coding
**Experience of violence**			
Missed school due to feeling unsafe	During the past 30 days, on how many days did you not go to school because you felt you would be unsafe at school or on your way to or from school?	0 days, 1 day, 2 or 3 days, 4 or 5 days, or ≥6 days	Yes (1 day, 2 or 3 days, 4 or 5 days, or ≥6 days) versus no (0 days)
Threatened or injured with a weapon at school	During the past 12 months, how many times has someone threatened or injured you with a weapon such as a gun, knife, or club on school property?	0 times, 1 time, 2 or 3 times, 4 or 5 times, 6 or 7 times, 8 or 9 times, 10 or 11 times, or ≥12 times	Yes (1 time, 2 or 3 times, 4 or 5 times, 6 or 7 times, 8 or 9 times, 10 or 11 times, or ≥12 times) versus no (0 times)
Bullied at school	During the past 12 months, have you ever been bullied on school property?	Yes or no	Yes versus no
Electronically bullied	During the past 12 months, have you ever been electronically bullied? (Count being bullied through texting, Instagram, Facebook, or other social media.)	Yes or no	Yes versus no
**Mental health**			
Frequent mental distress during the past <30 days	During the past 30 days, how often was your mental health not good? (Poor mental health includes stress, anxiety, and depression.)	Never, rarely, sometimes, most of the time, or always	Yes (most of the time or always) versus no (never, rarely, or sometimes)
Experienced persistent feelings of sadness or hopelessness during the past 12 months	During the past 12 months, did you ever feel so sad or hopeless almost every day for 2 weeks or more in a row that you stopped doing some usual activities?	Yes or no	Yes versus no
**Suicidal thought or behavior**			
Seriously considered suicide during the past 12 months	During the past 12 months, did you ever seriously consider attempting suicide?	Yes or no	Yes versus no
Made a suicide plan during the past 12 months	During the past 12 months, did you make a plan about how you would attempt suicide?	Yes or no	Yes versus no
Attempted suicide during the past 12 months	During the past 12 months, how many times did you actually attempt suicide?	0 times, 1 time, 2 or 3 times, 4 or 5 times, or ≥6 times	Yes (1 time, 2 or 3 times, 4 or 5 times, or ≥6 times) versus no (0 times)
Had a suicide attempt treated by a doctor or nurse during the past 12 months	If you attempted suicide during the past 12 months, did any attempt result in an injury, poisoning, or overdose that had to be treated by a doctor or nurse?	Yes, no, or I did not attempt suicide during the past 12 months	Yes versus no (no or I did not attempt suicide during the past 12 months)
**School connectedness**			
Felt close to others at school	Do you agree or disagree that you feel close to people at your school?	Strongly agree, agree, not sure, disagree, or strongly disagree	Yes (strongly agree or agree) versus no (strongly disagree, disagree, or not sure)
**Housing**			
Experienced unstable housing	During the past 30 days, where did you usually sleep?	In my parent's or guardian's home; in the home of a friend, family member, or other person because I had to leave my home or my parent or guardian cannot afford housing; in a shelter or emergency housing; in a motel or hotel; in a car, park, campground, or other public place; or I do not have a usual place to sleep or somewhere else	Yes (in the home of a friend, family member, or other person because I had to leave my home or my parent or guardian cannot afford housing; in a shelter or emergency housing; in a motel or hotel; in a car, park, campground, or other public place; or I do not have a usual place to sleep) versus no (in my parent's or guardian's home or somewhere else)

**TABLE 2:** Demographic characteristics stratified by transgender identity among high school students — Youth Risk Behavior Survey, United States, 2023*

Characteristic	Gender identity^†^			*t*-test p value^§^		
	Cisgender (n = 16,986)^¶^	Transgender (n = 612)^¶^	Questioning (n = 428)^¶^	Cisgender versus transgender	Cisgender versus questioning	Transgender versus questioning
Total	% (95% CI)**	% (95% CI)**	% (95% CI)**			
	94.5 (93.6–95.3)	3.3 (2.8–4.0)	2.2 (1.8–2.7)			
**Sex^††^**						
Female	47.5 (45.2–49.8)	64.2 (57.2–70.5)	64.3 (57.9–70.5)	<0.0001	<0.0001	0.9693
Male	52.5 (50.2–54.8)	35.8 (29.5–42.8)	35.7 (29.7–42.1)	<0.0001	<0.0001	0.9693
**Race or ethnicity** ^§§^						
American Indian or Alaska Native	0.3 (0.2–0.5)	——^¶¶^	——	——	——	——
Asian	4.3 (2.8–6.5)	——	——	——	——	——
Black or African American	13.8 (9.4–19.7)	5.4 (3.0–9.5)	11.8 (7.0–19.2)	0.0003	0.4008	0.0144
Native Hawaiian or other Pacific Islander	0.4 (0.1–1.0)	——	——	——	——	——
White	48.3 (41.3–55.3)	64.0 (52.7–73.9)	46.4 (35.7–57.6)	0.0022	0.7404	0.0015
Hispanic or Latino	26.8 (22.2–32.0)	21.5 (13.2–33.0)	27.7 (17.6–40.7)	0.2458	0.8648	0.2298
Multiracial	6.1 (4.4–8.5)	5.3 (2.6–10.5)	9.2 (5.1–16.1)	0.5039	0.2368	0.1898
**Grade **						
9	26.5 (24.3–28.8)	24.4 (19.3–30.4)	20.6 (16.4–25.6)	0.4702	0.0123	0.2403
10	25.8 (24.0–27.6)	24.3 (19.2–30.2)	29.0 (22.0–37.1)	0.5972	0.3779	0.2912
11	24.1 (22.1–26.3)	25.4 (19.9–32.0)	33.4 (24.8–43.4)	0.6422	0.0529	0.0729
12	23.6 (21.4–26.0)	25.9 (19.7–33.2)	17.0 (12.2–23.1)	0.5066	0.0133	0.0281
Sexual identity***						
Heterosexual	79.4 (77.3–81.3)	8.7 (4.9–15.0)	7.5 (3.4–15.8)	<0.0001	<0.0001	0.5719
Gay or lesbian	3.1 (2.7–3.7)	25.0 (19.4–31.5)	15.5 (10.1–23.1)	<0.0001	0.0003	0.0491
Bisexual	10.5 (9.4–11.8)	26.5 (20.7–33.3	33.4 (26.7–40.9)	<0.0001	<0.0001	0.1725
Questioning	4.1 (3.5–4.7)	7.0 (4.1–11.5)	20.4 (15.0–27.1)	0.0933	<0.0001	0.0004
Describe in some other way	2.9 (2.5–3.5)	32.8 (26.4–39.9)	23.2 (16.9–31.0)	<0.0001	<0.0001	0.0232

* N = 20,103 respondents. The total number of students answering each question varied. Data may be missing because 1) the question did not appear in that student’s questionnaire, 2) the student did not answer the question, or 3) the response was set to missing because of an out-of-range response or logical inconsistency. Percentages in each category are calculated on the known data.

^†^ Transgender identity was categorized as transgender for those who responded, “Yes, I am transgender,” to the question, “Some people describe themselves as transgender when their sex at birth does not match the way they think or feel about their gender. Are you transgender?” Cisgender students are those who responded, “No, I am not transgender.” Questioning students are those who responded, “I am not sure if I am transgender.”

^§^ Pairwise *t*-test analysis for difference in student characteristics between gender identity groups (p<0.05).

^¶^ Unweighted sample size.

** Weighted prevalence estimate.

^††^ Sex is reported for transgender and questioning students for descriptive purposes. Because the sex question does not specify sex assigned at birth, there may be differences in interpretation among transgender students. For this reason, transgender students are not categorized by sex for other analyses.

^§§^ Persons of Hispanic or Latino origin might be of any race but are categorized as Hispanic; all racial groups are non-Hispanic.

^¶¶^ Dashes indicate estimates and p values not available because denominator sample sizes are <30.

*** Students who responded, “I don’t know what this question is asking” were excluded from analysis of sexual identity.

### Analysis

All prevalence estimates used Taylor series linearization. The prevalence of transgender, cisgender, and transgender questioning students were estimated for students overall and by sex, grade, race and ethnicity, and sexual identity. Differences in demographic characteristics by transgender identity were assessed using pairwise *t*-tests. Presenting the prevalence estimate of each health behavior and experience for transgender and questioning students permitted description of the effects of adverse health challenges that they faced separately from cisgender students. Adjusted prevalence estimates of health behaviors and experiences stratified by cisgender male, cisgender female, transgender, and questioning students were calculated using logistic regression with predicted marginals, controlling for underlying differences by race and ethnicity and grade. Prevalence estimates with denominators <30 were considered statistically unreliable and therefore were suppressed (*6*). Differences in adjusted prevalence by transgender identity were assessed through pairwise *t*-test analysis. Differences in results were considered statistically significant at p<0.05. Analyses were conducted using SAS-callable SUDAAN (version 11.0.3; RTI International), accounting for complex survey design and weighting.

## Results

### Demographic Characteristics

Approximately 3.3% of high school students identified as transgender, and 2.2% reported questioning if they were transgender (Table 2). Most students (94.5%) did not identify as transgender or questioning. Differences in demographic characteristics were observed by transgender identity. Approximately half of cisgender students reported female sex (47.5%). Approximately two thirds of transgender or questioning students reported female sex (64.2% and 64.3%, respectively). Differences in race and ethnicity by transgender identity were observed. A lower proportion of transgender students identified as Black and higher proportion identified as White compared with cisgender or questioning students. In addition, for questioning students, differences in grade distribution were observed.

Differences in sexual identity were observed by transgender identity. Most cisgender students reported their sexual identity as heterosexual (79.4%), whereas only 8.7% of transgender students and 7.5% of questioning students identified as heterosexual. Transgender questioning students had a higher prevalence of questioning their sexual identity (20.4%) than both cisgender and transgender students (4.1% and 7.0%, respectively). The prevalence of students who described their sexual identity in some other way was greatest among transgender students (32.8%), followed by transgender questioning students (23.2%), with only 2.9% of cisgender students identifying as such.

### Health Behaviors and Experiences

Unadjusted prevalence estimates reflect higher prevalence of adverse health behaviors and experiences for transgender and questioning students (Supplementary Table, https://stacks.cdc.gov/view/cdc/159811). Because of the differences in race and ethnicity and grade when comparing transgender, questioning, and cisgender students, adjusted prevalence estimates are presented (Table 3). Transgender and questioning students had the highest prevalence of experiencing violence, poor mental health, suicidal thoughts and behaviors, and unstable housing, and the lowest prevalence of school connectedness compared with cisgender students (Figure). Approximately one fourth of transgender and questioning students missed school because of feeling unsafe in the past 30 days (25.3% and 26.4%, respectively) compared with 8.5% of cisgender male students and 14.9% of cisgender female students. Being bullied at school in the past 12 months was the most prevalent experience of violence for all four gender identity categories, but a higher prevalence of bullying was reported by transgender (40.1%) and questioning (39.9%) students than cisgender female (20.3%) and cisgender male students (14.8%).

**TABLE 3:** Prevalence estimates of experiences of violence, poor mental health, suicidal thoughts and behaviors, school connectedness, and unstable housing by gender identity among high school students — Youth Risk Behavior Survey, United States, 2023*

Health behavior and experience	Gender identity^†^			
	Cisgender male (n = 8,643)^§^	Cisgender female (n = 8,284)^§^	Transgender (n = 612)^§^	Questioning (n = 428)^§^
	% (95% CI)^¶^	% (95% CI)^¶^	% (95% CI)^¶^	% (95% CI)^¶^
**Experience of violence**				
Skipped school due to feeling unsafe there during the past 30 days	8.5 (6.5–10.9)	14.9 (12.0–18.3)**	25.3 (18.0–34.2)**^,††^	26.4 (19.3–35.0)**^,††^
Threatened or injured with a weapon at school during the past 12 months	8.5 (7.4–9.8)	8.0 (6.7–9.5)	13.4 (9.1–19.3)^††^	19.6 (13.6–27.3)**^,††^
Bullied at school during the past 12 months	14.8 (13.6–16.1)	20.3 (18.1–22.8)**	40.1 (32.7–48.0)**^,††^	39.9 (32.4–47.9)**^,††^
Electronically bullied during the past 12 months	10.6 (9.5–11.8)	19.1 (17.3–21.0)**	31.3 (25.1–38.2)**^,††^	30.7 (25.4–36.5)**^,††^
**Mental health**				
Poor mental health during the past 30 days	17.8 (16.3–19.4)	37.8 (35.6–40.2)**	64.9 (56.6–72.4)**^,††,§§^	53.3 (45.2–61.2)**^,††^
Persistent feelings of sadness or hopelessness during the past 12 months	26.0 (24.5–27.6)	50.5 (48.0–52.9)**	71.9 (64.0–78.6)**^,††^	68.9 (62.4–74.7)**^,††^
**Suicidal thoughts and behavior**				
Seriously considered attempting suicide during the past 12 months	12.1 (10.7–13.5)	24.0 (22.1–26.0)**	52.9 (46.0–59.7)**^,††^	44.9 (39.4–50.5)**^,††^
Made a suicide plan during the past 12 months	10.4 (9.4–11.4)	19.2 (17.4–21.2)**	39.8 (34.7–45.2)**^,††^	38.1 (32.0–44.6)**^,††^
Attempted suicide during the past 12 months	5.3 (4.3–6.4)	11.0 (9.8–12.3)**	25.9 (20.9–31.7)**^,††^	25.8 (19.6–33.1)**^,††^
Had a suicide attempt treated by a doctor or nurse during the past 12 months	1.0 (0.6–1.5)	2.6 (2.1–3.1)**	10.3 (6.4–16.4)**^,††^	3.7 (1.5–9.3)^††^
**School connectedness**				
Felt close to others at school	61.9 (58.9–64.7)	50.7 (47.9–53.5)**	36.6 (29.4–44.4)**^,††,§§^	45.9 (38.7–53.3)**
**Housing**				
Unstable housing during the past 30 days	2.1 (1.6–2.7)	1.8 (1.2–2.7)	10.7 (5.1–21.2)**^,††^	10.0 (3.1–27.4)

* N = 20,103 respondents. Numbers might not sum to totals because of missing data.

^†^ Transgender identity was categorized as transgender for those who responded, “Yes, I am transgender” to “Some people describe themselves as transgender when their sex at birth does not match the way they think or feel about their gender. Are you transgender?” Questioning students are those who responded, “I am not sure if I am transgender.” Cisgender students are those who responded, “No, I am not transgender.” Because the sex question does not specify sex assigned at birth, there might be differences in interpretation among transgender students. For this reason, only cisgender students are further disaggregated by sex.

^§^ Unweighted sample size.

^¶^ Weighted, model-adjusted prevalence estimate. Logistic regression models adjusted for race and ethnicity and grade with specifications for predicted marginal proportions to produce adjusted prevalence estimates for each health behavior and experience.

** Significantly different from cisgender male students as determined by pairwise *t*-test analysis (p<0.05).

^††^ Significantly different from cisgender female students as determined by pairwise *t*-test analysis (p<0.05).

^§§^ Significantly different from questioning students as determined by pairwise *t*-test analysis (p<0.05).

**FIGURE:**
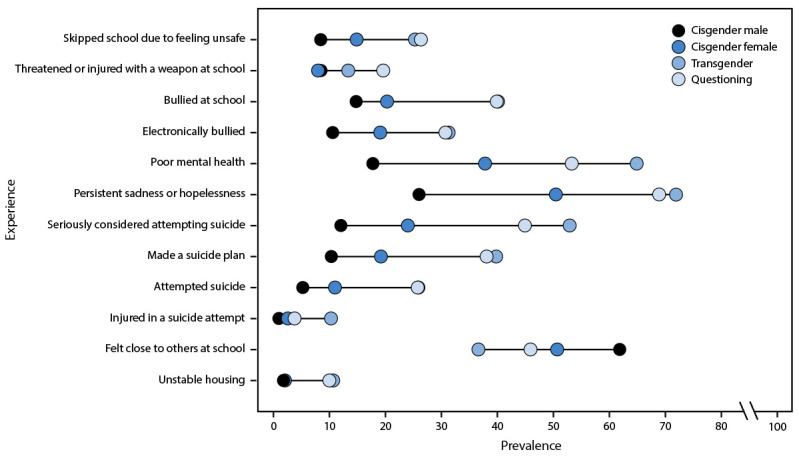
Adjusted prevalence estimates* of experiences of violence, poor mental health, suicidal thoughts and behaviors, school connectedness, and unstable housing by transgender identity among high school students — Youth Risk Behavior Survey, United States, 2023 * Logistic regression models adjusted for race and ethnicity and grade with specifications for predicted marginal proportions to produce adjusted prevalence estimates for each health behavior and experience.

Similar differences in mental health and suicidal thoughts and behaviors were found for transgender and questioning students. Among transgender students, 64.9% reported poor mental health in the past 30 days and 71.9% reported persistent sadness or hopelessness in the past 12 months. Questioning students had a similarly high prevalence of these outcomes (53.3% and 68.9%, respectively). Cisgender females had the next highest prevalence of poor mental health (37.8%) and persistent feelings of sadness or hopelessness (50.5%), with cisgender males having the lowest prevalence of both outcomes (17.8% and 26.0%, respectively). Approximately half of transgender students (52.9%) and 44.9% of questioning students seriously considered attempting suicide in the past year, compared with 24.0% of cisgender females and 12.1% of cisgender males. Approximately one fourth of transgender and questioning students attempted suicide in the past year (25.9% and 25.8%, respectively) compared with 11.0% of cisgender females and 5.3% of cisgender males.

Transgender students reported the lowest prevalence of feeling close to others at school (36.6%), followed by questioning (45.9%) and cisgender female students (50.7%), with cisgender male students reporting the highest prevalence (61.9%). Transgender students had a higher prevalence of experiencing unstable housing in the past 30 days (10.7%) than questioning (10.0%), cisgender male (2.1%), and cisgender female students (1.8%).

## Discussion

This study presents the first nationally representative prevalence estimates of transgender identity and questioning transgender identity among U.S. high school students, building on previous research among states and local urban school districts that have included the transgender identity item in their YRBSs since 2017 (*1*). Analysis of 18 states’ 2021 YRBS data found similar prevalence of transgender identity and similar distributions across demographic characteristics of transgender and questioning students (*7*).

This study found that transgender and questioning students face a higher prevalence of experiencing violence, poor mental health, suicidal thoughts and behaviors, and unstable housing and a lower prevalence of school connectedness compared with their cisgender peers. Approximately 40% of transgender and questioning students were bullied at school. Approximately 26% of transgender and questioning students attempted suicide in the past year, compared with approximately 5% of cisgender males. The prevalence of unstable housing was highest among transgender students (10.7%) and lowest among cisgender females (1.8%). The disparities identified in this study are consistent with those from previous studies using state YRBS, clinical, and convenience samples (*1*,*8*). Previous research using 2017 and 2019 state YRBS data demonstrated that the prevalence of unstable housing was more than seven times higher among transgender and questioning students combined, who were also three times more likely to be living “on the streets” (i.e., in a car, park, campground, or other public place) when experiencing unstable housing, compared with cisgender students (*9*).

Minority stress theory and the gender minority stress framework (*10*) can be applied to understand the factors that perpetuate these disparities: Transgender and questioning persons experience stigma, discrimination, and social marginalization related to their gender as a result of institutionalized social norms that privilege cisgender persons. The accumulation of stressors, including internalization of stigmatized attitudes, expectations of rejection, and experiences of discrimination and violence, can increase the likelihood that transgender and questioning persons experience poor mental health and lead to disparities in health and well-being. Transgender and questioning students might face stressors in their family life (e.g., adverse childhood experiences, parental rejection, and misgendering) and school life (e.g., bullying, violence, misgendering by peers or teachers, and being denied access to activities aligned with their gender identity) that might increase their risk for poor mental health (*8*). Furthermore, transgender students of color might face additional marginalization related to their race or ethnicity. According to the GLSEN 2021 National School Climate Survey, approximately 80% of lesbian, gay, bisexual, transgender, and queer/questioning (LGBTQ+) students (K–12) experienced verbal, physical, or sexual harassment or assault at school, and approximately half of LGBTQ+ students of color experienced victimization related to race and ethnicity (*11*).

The structural and interpersonal discrimination, including family rejection, faced by transgender students puts this population at increased risk for experiencing unstable housing (*9*). Transgender students might experience discrimination, harassment, and assault among foster, shelter, and other social service providers that make this population less likely to be sheltered when experiencing unstable housing, compounding their vulnerability to experiences of violence, poor mental health, and suicidal thoughts and behaviors (*9*).

The findings in the report demonstrate that transgender and questioning students experience more violence, less school connectedness, more unstable housing, poorer mental health, and more suicidal thoughts and behaviors than their cisgender peers, underscoring the need for interventions to create safe and supportive environments for transgender and questioning students. Having supportive families and peers, feeling connected to family and school, having affirmed name and pronouns used consistently by others, and having a sense of pride of identity are protective factors for transgender students that buffer the effects of minority stressors and promote better mental health (*8*).

### Intervention Opportunities

Schools are in a unique position to create safe and supportive environments, free from violence and bullying, for all students, including transgender and questioning students. Violence, poor mental health, and suicide are not caused by any single factor, and prevention will not be achieved by any single strategy. However, strategies that create safe and supportive environments inclusive of transgender students and promote school connectedness can improve the health and well-being of transgender students across a range of outcomes. Evidence supports the association of CDC’s What Works in Schools (WWIS) approach (https://www.cdc.gov/healthyyouth/whatworks/what-works-overview.htm) with reductions in experiences of violence, poor mental health, and suicidal thoughts and behaviors among high school students (*12*). WWIS supports districts and schools to implement quality and inclusive health education, connect students to health services, and foster safe and supportive school environments. In particular, school connectedness and activities to promote safe and supportive environments are associated with decreased odds of experiencing violence, poor mental health, and suicidal thoughts and behaviors among high school students (*13*). Activities that are inclusive of LGBTQ+ students are associated with decreases in the odds of these experiences among all students regardless of sexual identity (*14*). Inclusive activities might involve implementing genders and sexualities alliances (student-led clubs offering a means for students with LGBTQ+ identities and allies to gather and provide support), providing professional development to educators and school staff members on supporting students with LGBTQ+ identities, providing mental health and other health service referrals that are inclusive of students with LGTBQ+ identities, and implementing policies that are inclusive of students with LGBTQ+ identities. To date, the WWIS approach has not been evaluated specifically among transgender and questioning students. Further research is necessary; however, the possibility of school supports as health enhancing for transgender and questioning students is promising.

CDC’s Dating Matters (https://www.cdc.gov/intimate-partner-violence/php/datingmatters/index.html) is an evidence-based teen dating violence prevention model that educates adolescents on healthy relationships of certain types, including relationships with family and friends, and is effective for reducing risk for both experiencing and perpetrating violence and engaging in substance use. Dating Matters has been adapted to create A Guide to Healthy, Safe Relationships for LGBTQ+ Youth (https://vetoviolence.cdc.gov/apps/dating-matters-toolkit/static/media/Dating_Matters_LGTBQ%20Guide_Youth_v5a_508.fde67eab.pdf), a tailored resource that provides information on healthy relationships specific to the unique needs and experiences of students with LGBTQ+ identities. CDC’s Suicide Prevention Resources for Action (https://www.cdc.gov/suicide/resources/prevention.html) identifies strategies for a comprehensive approach to suicide prevention that addresses the multiple factors associated with suicide risk. The implementation of school-based strategies and community-based supports can serve as the foundation for effective youth suicide prevention. Schools can create safe and supportive environments and promote connectedness by teaching coping and problem solving, providing gatekeeper training to peers, teachers, and other adults at school, and implementing mental health support (the term “gatekeeper” refers to persons trained to identify people at risk for suicide and to respond effectively by facilitating referrals to treatment and support services https://www.cdc.gov/suicide/pdf/preventionresource.pdf). CDC’s Comprehensive Suicide Prevention Program (https://www.cdc.gov/suicide/programs/csp.html) funds 24 programs to implement and evaluate a comprehensive public health approach to suicide prevention, with a special focus on populations disproportionately affected by suicide, including transgender and questioning students.

The McKinney-Vento Homeless Assistance Act^†^ (MVA) is a Federal law that authorizes services that allow students experiencing unstable housing to enroll, attend, and achieve success in school. Certain MVA programs provide training and support for referrals to school- and community-based programs for family counseling, adolescent health and mental health care, and LGBTQ+ programs supported by student-led groups including genders and sexualities alliances (*15*). Schools can play a pivotal role in supporting transgender and questioning students experiencing unstable housing by implementing and connecting students with such MVA-funded programs tailored to the needs of this population.

## Limitations

General limitations for the YRBS are available in the overview report of this supplement (*6*). The findings in this report are subject to at least four additional limitations. First, because of the low number of AI/AN, Asian, and NH/OPI students identifying as transgender, data among these groups were suppressed; however, continued collection of transgender identity in the national YRBS will allow for aggregating data across cycles to achieve larger sample sizes. Second, the survey question assessing sex on the YRBS does not specify sex assigned at birth. Transgender students might not respond to the sex survey question consistently as their sex assigned at birth or gender identity and therefore this analysis could not further disaggregate transgender students. Third, sex is used to calculate sample weights used in analyses, which might therefore be inaccurate for transgender students. Population-based surveys such as YRBS are needed to establish the prevalence of transgender and questioning adolescents in the United States so that future surveys might be able to incorporate transgender identity into survey weights. Finally, students who responded, “No, I am not transgender,” to the transgender identity item were assumed to be cisgender, but students with this response also might have a gender identity other than cisgender that might not be recorded by transgender identity (e.g., nonbinary, genderfluid, and agender).

## Future Directions

More research is needed in describing experiences among transgender and questioning students by race and ethnicity and by more specific measures of gender identity, such as differences for nonbinary students, transgender girls, and transgender boys. Further research is needed on health behaviors and experiences not analyzed in the present study, including adverse childhood experiences and social media usage, which might relate to adolescent mental health. In addition, continued research on the school-based strategies that can best support transgender students is needed to tailor existing strategies, develop clear guidance for schools and families, and identify innovative strategies that achieve equity for transgender and questioning students.

## Conclusion

These results provide insight into the challenges faced by transgender and questioning students and provide much needed context for ongoing discussions about how best to support and protect transgender and questioning students. These are the first nationally representative data on transgender and questioning students. Their school environments are neither as safe nor as supportive as they are for their cisgender peers. That transgender and questioning students are more likely to experience poor mental health and suicidal thoughts and behaviors than their cisgender peers is concerning. Tools exist to improve the safety and supportiveness of schools, and research demonstrates that when schools make steps to implement inclusive policies and practices, the mental health of all students improves. More effort is necessary to ensure that the health and well-being of students who are socially marginalized is prioritized.

## Footnotes

* 45 C.F.R. part 46.114; 21 C.F.R. part 56.114.

† 42 USC chapter 119, subchapter VI, part B: Education for homeless children and youths. https://uscode.house.gov/view.xhtml?path=/prelim%40title42/chapter119/subchapter6/partB&edition=prelim
